# Altered expression profile of BAFF receptors on peripheral blood B lymphocytes in Graves’ disease

**DOI:** 10.1186/s12902-021-00752-3

**Published:** 2021-04-29

**Authors:** Xin Wang, Jinhui Huang, Aixia Zhang, Chen Fang, Qi Ma, Pengjun Jiang

**Affiliations:** 1 Department of Endocrinology, Jiangsu Province Hospital of TCM/the Affiliated Hospital of Nanjing University of Chinese Medicine, 210029 Nanjing, China; 2 School of Pharmacy, Nanjing Medical University, 211166 Nanjing, China; 3 Clinical Nutrition Department, Second affiliated Hospital of Soochow University, 215004 Suzhou, China; 4 Department of Ultrasound, Second affiliated Hospital of Soochow University, 215004 Suzhou, China; 5 Department of Hematology, Jiangsu Province Hospital of TCM/the Affiliated Hospital of Nanjing University of Chinese Medicine, 210029 Nanjing, China

**Keywords:** Graves’ disease, Hashimoto’s thyroiditis, BAFF, BR3, TACI

## Abstract

**Background:**

B lymphocyte activating factor (BAFF) is a growth factor regulating B lymphocytes survival and maturation. Serum BAFF levels were elevated in patients affected with autoimmune thyroid diseases (AITD), including Graves’ disease (GD) and Hashimoto’s thyroiditis (HT). The aim of this study is to explore the association of expression levels of BAFF and its receptors with AITD.

**Methods:**

Fifty-two GD patients, 39 Hashimoto’s thyroiditis (HT) patients and 23 healthy controls (HC) were recruited in this study. Serum BAFF levels were measured by ELISA. Expression of BAFF receptors, including BAFF receptor 3 (BR3) and transmembrane activator and calcium-modulating and cyclophilin ligand interactor (TACI), on B lymphocytes were analyzed by flowcytometry. Effects of steroids on serum BAFF levels and expression of BR3 and TACI were also observed in 10 patients with Graves’ orbitopathy (GO) receiving steroids therapy.

**Results:**

Serum BAFF levels were significantly elevated from 0.93 ± 0.24 ng/ml in HC to 1.18 ± 0.33 ng/ml in GD (*P* = 0.0027) and 1.02 ± 0.24 ng/ml in HT (*P* = 0.0331). BR3 expression on peripheral B lymphocytes were elevated in GD (mean MFI: 4.52 ± 2.06 in GD vs. 3.00 ± 0.87 in HC, *P* = 0.0015), while TACI expression on peripheral B lymphocytes were decreased in GD without significance (mean MFI: 7.96 ± 4.06 in GD vs. 9.10 ± 3.37 in HC, *P* = 0.1285). Expression of BR3 and TACI was not changed significantly in HT patients. Steroids significantly suppressed serum BAFF concentrations (from 1.18 ± 0.27 ng/ml to 0.97 ± 0.10 ng/ml, *P* = 0.0364) and BR3 expression in GO patients (mean MFI from 6.26 ± 4.91 to 4.05 ± 1.58, *P* = 0.0083).

**Conclusions:**

Altered expression of BAFF and its receptor may mediate the autoimmunity in GD. Restoring the normal expression profile of receptors for BAFF could be a new strategy to treat GD.

## Background

Graves’ disease (GD) is an autoimmune disease characterized by the presence of the thyrotropin receptor specific autoantibody (TRAb) [1]. The TRAb binds to the thyrotropin receptor with agonistic properties and stimulates the over production of thyroid hormone [1]. The production of autoantibodies is a result of aberrant activation of autoreactive B lymphocytes. Under normal conditions, these autoreactive B lymphocytes are eliminated or in an inactivated state which was called anergy. However, in autoimmune diseases, the excess production of B cell activation factor (BAFF, also known as B lymphocytes stimulator, Blys) supports the survival and activation of these defective autoreactive B lymphocytes [2, 3].

BAFF provides B lymphocytes with essential survival signals [2]. Because its ability to rescue low-affinity autoreactive transitional B lymphocytes at tolerance checkpoints and promote their maturation [2, 3], BAFF has long been linked to autoimmune diseases. Over expression of BAFF in mice induced a dramatic expansion of activated autoreactive B lymphocytes and autoantibody production [4]. In humans, serum BAFF levels are elevated in autoimmune diseases including systemic lupus erythematosus (SLE) [5], autoimmune hepatitis [6] and primary Sjögren’s syndrome [7].

Accumulated studies suggest BAFF is involved in the pathogenesis of autoimmune thyroid diseases (AITD). BAFF gene polymorphisms have been linked to the susceptibility to GD [8, 9]. Serum BAFF levels are elevated in patients affected with AITD [10–12]. BAFF was also shown to affect the occurrence of thyroid autoimmunity in chronic hepatitis C patients receiving interferon alpha therapy [13]. Blocking BAFF activity by soluble BAFF receptor-Fc fusion protein relieved hyperthyroidism in a murine model [14]. Finally, steroids improve Graves’ orbitopathy (GO) through inhibiting BAFF secretion [12].

BAFF has three cell surface receptors: BAFF receptor 3 (BR3, CD268, or TNFRSF13C) [15], transmembrane activator and calcium-modulating and cyclophilin ligand interactor (TACI, CD267, or TNFRSF13B) [16] and B cell maturation antigen (BCMA or TNFRSF17) [17]. The three receptors for BAFF have different expression profiles based on B cell developmental stages [3, 18]. As the dominant BAFF receptor, BR3 is expressed on almost all B lymphocytes that express functional B cell receptor (BCR), including naïve B cells, marginal zone B cells and switched memory B cells. TACI is mainly expressed by CD27^+^ memory B cells and a small part of plasma cells, while BCMA is expressed restrictedly on plasmablasts and plasma cells. Campi et al. found that expression of BAFF and BR3 in thyroid infiltrating lymphocytes (TIL) in AITD was higher than that of multinodular goiter [19]. However, it is not clear how the expression of BAFF receptors on peripheral blood B lymphocytes changes in the context of GD.

In the present study, to investigate whether the expression of different BAFF receptors on the peripheral blood cells was associated with the autoimmunity of GD, we measured the expression of two BAFF receptors, BR3 and TACI, on the peripheral blood B lymphocytes in GD patients. Furthermore, effects of steroids on the expression of BAFF receptors in patients affected with GO were also observed.

## Methods

### Power calculation and patients

Power was calculated by R package. We set the Type I Error = 0.05 and the Type II Error = 0.2. Based on the data of our initial assay, at least 14 subjects should be enrolled. Indeed 52 newly diagnosed GD patients, 39 HT patients and 23 healthy controls (HC) were recruited from Jiangsu Province Hospital of TCM/the Affiliated Hospital of Nanjing University of Chinese Medicine. All the GD patients were in hyperthyroidism state. In the HT group, 29 patients had normal thyroid function, 5 patients were with subclinical hypothyroidism and the remaining 5 patients were clinical hyperthyroidic.

### Therapy

All GD patients received methimazole therapy. Initial methimazole dosage was determined by serum free tetraiodothyronine concentration according to the American thyroid association (ATA) guideline for management for hyperthyroidism [20]. Ten patients with clinical activity score (CAS) above 3/7 at the first examination were diagnosed with active GO according to the European Group of Graves’ Orbitopathy (EUGOGO) classification system [21]. These patients underwent methylprednisolone iv injection in addition to administration of methimazole. Methylprednisolone was administered 500 mg weekly in the first 6 weeks and 250 mg weekly in the following 6 weeks as Zhu et al.. described [22].

### Thyroid function and thyroid autoantibodies assay

Thyroid function, including serum thyroid stimulating hormone (TSH), free thyroxine (FT4) and free triiodothyronine (FT3) levels, and TRAb titers, were measured by Roche Elecys electrochemiluminescence immunoassay (ECL) kit. Thyroid peroxidase autoantibody (TPOAb) was assayed by ECL kit from Beckman Coulter.

### BAFF ELISA

Serum BAFF concentrations were determined by the Quantikine ELISA kit (R&D systems, DBLYS0B). The sensitivity of the kit is 2.68 pg/ml, and the intra-assay coefficient of variation is 5.6 %. All the samples were measured duplicate. Blank and serially diluted recombinant BAFF (from 4,000pg/ml to 62.5pg/ml) were tested simultaneously to establish standard curve. Concentration of BAFF in each sample was calculated according to the formula deducted from the standard curve.

### Cell staining and flow cytometry

Peripheral blood mononuclear cells (PBMC) were isolated from 5ml fresh anti-coagulated blood by lymphoprep (StemCell Technologies) and blocked with FcR blocking buffer (Miltenyi Biotec, cat number: 130-059-901) at 4˚C for 10 min in the dark. Cells were then stained with fluorescein isothiocyanate (FITC) conjugated anti human CD19 (thermofisher, cat number: 11-0199-42) with either phycoerythrin (PE) conjugated anti human BR3 (thermofisher, cat number: 12-9117-42) or PE conjugated anti human TACI (thermofisher, cat number: 12-9217-42) at 4˚Cfor 30 min in the dark. Cells were acquired on a Navios flow cytometer (Beckman, U.S.). Data were analyzed by using Kaluza Flow Cytometry Analysis Software Version 2.0(Beckman, U.S.). For each test, at least 50,000 PBMCs were acquired. Mean fluorescent intensity (MFI) of eBR3 and TACIonCD19^+^ cells were determined. Data are represented as Mean ± SD.

### Statistics

Statistical analysis was performed by Graphpad Prism Version 6.0. One-way ANOVA and t-test were applied to compare serum BAFF concentrations, MFI of BR3 or TACI among groups. Correlations were analyzed by Spearman’s rank correlation tests. A *P* value less than 0.05 was considered statistically significant.

## Results

### 1. Subjects characteristics

Characteristics of the patients and controls were listed in Table 1.

**Table 1:** Characteristics of the subjects in the study

Group	n	Gender M/F	Age	TRAb (IU/L)	TPOAb (IU/L)	TSH (mIU/L)	FT3 (pg/ml)	FT4 (ng/dl)	Methimazole (mg)
GD	52	18/34	36.46 ± 11.53	11.98 ± 20.53	179.18 ± 253.26	0.16 ± 0.41	6.17 ± 4.97	1.88 ± 1.35	14.71 ± 8.37
HT	39	12/27	37.89 ± 15.59	N.A.	221.62 ± 317.05	5.55 ± 9.80	3.2 0 ± 0.67	0.86 ± 0.17	N.A.
HC	23	12/11	39.96 ± 12.14	< 1.75	< 3.5	N.A.	N.A.	N.A.	N.A.

*TRAb* thyrotropin receptor autoantibody; *TPOAb* thyroid peroxidase autoantibody; *TSH* thyroid stimulating hormone; *FT3* free triiodothyronine; *FT4* free thyroxine; *GD* Graves’ disease; *HT* Hashimoto’s thyroiditis; *FT4* free thyroxine

### 2. Elevated serum BAFF levels in patients affected with AITD

**Fig. 1:**
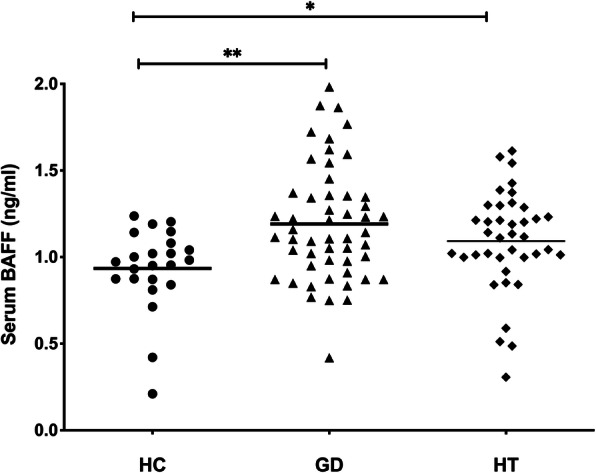
Increased serum BAFF levels in AITD patients. Serum BAFF levels were elevated in GD patients (1.183 ± 0.334 ng/ml) and HT patients (1.02 ± 0.24), comparing with healthy controls (0.934 ± 0.238 ng/ml). AITD: autoimmune thyroid disease; HC: healthy controls; GD: Graves’ disease; HT: Hashimoto’s thyroiditis; Bars: mean BAFF levels; ns: not significant; *: vs. HC, *P* < 0.05, **: vs. HC, *P* < 0.01

Serum BAFF levels were elevated in autoimmune diseases including GD in previous studies. To confirm these findings, we measured serum BAFF concentrations in both AITD patients and healthy controls by ELISA. As shown in Fig. 1, serum BAFF levels were significantly higher in patients with either GD (1.18 ± 0.33ng/ml, *P* = 0.0027) or HT (1.02 ± 0.24, *P* = 0.0331)than those in healthy controls (0.93 ± 0.24ng/ml, Fig. 1). There was no difference in BAFF concentrations between euthyroid patients and hypothyroid patients in HT group (data were not shown).

Despite previous studies showed serum BAFF levels were associated with activity of autoimmune, there were inconsistent results on the correlations between BAFF expression levels and autoantibodies titers. We thus investigated whether serum BAFF levels correlated with either thyroid function or thyroid autoantibodies titers in AITD patients. As shown in Table 2, serum BAFF levels positively correlated with serum free thyroxine concentrations (Spearman *r* = 0.2460, *P* = 0.0394), but not the TRAb titers (*P* = 0.4734) in GD patients. However, serum BAFF concentrations were associated with TPOAb titers in both GD (*R* = 0.2451, *P* = 0.0399, Table 2) and HT (*R* = 0.2983, *P* = 0.0325, Table 2). There was no correlation between sera BAFF levels and free thyroxine concentrations in HT (*P* = 0.2655, Table 2).

**Table 2:** Correlations between serum BAFF concentrations and thyroid autoantibodies and thyroid hormones in AITD

	r	*P*
TRAb in GD	0.147	0.4734
TPOAb		
GD	0.2451	0.0399
HT	0.2983	0.0325
FT4		
GD	0.2460	0.0394
HT	-0.1034	0.2655

*TRAb* thyrotropin receptor autoantibody; *TPOAb* thyroid peroxidase autoantibody; *GD* Graves’ disease; *HT* Hashimoto’s thyroiditis; *FT4* free thyroxine

### 3. Altered expression profile of receptors for BAFF on peripheral blood B lymphocytes in Graves’ disease

**Fig. 2:**
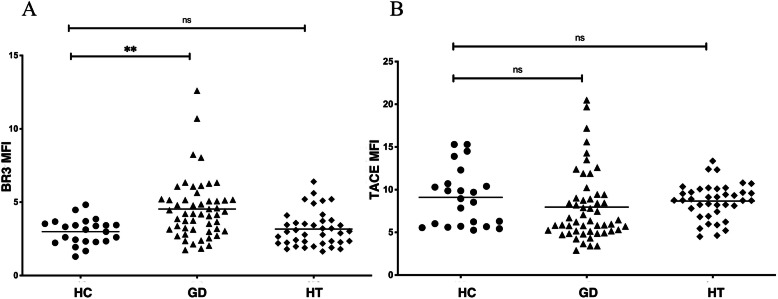
Altered expression profile of receptors of BAFF on peripheral blood B lymphocytes in GD. **a**: Elevated BR3 expression on peripheral blood B lymphocytes in GD patients. Mean MFI of BR3 on peripheral B lymphocytes in GD patients was 4.52 ± 2.06, higher than that in HC (3.00 ± 0.87), *P* = 0.0015; BR3 expression in HT patients was not increased; **b**: Trend of decreased TACI expression on peripheral B lymphocytes in GD patients. Mean MFI of TACI on peripheral B lymphocytes in GD patients was 7.96 ± 4.06, lower than that in HC (9.10 ± 3.37) without significance, *P* = 0.1285. TACI expression in HT was not decreased. MFI: mean fluorescence intensity. Bars: mean MFI. ns: not significant; **: vs. HC, *P* < 0.01

BAFF receptors includeBR3, TACI and BCMA. Because BCMA expression is restricted on plasma cells and plasmablasts, both of them exist at extremely low frequencies in peripheral blood, we only measured BR3 and TACI expression in this study. Most of CD19^+^ cells express BR3 while only part of B lymphocytes express TACI. The percentages of TACI-expressing B lymphocytes were comparable between healthy controls and GD patients (data were not shown). The expression levels of BR3 on peripheral blood B lymphocytes of GD patients were significantly higher than that of healthy controls (mean MFI: 4.52 ± 2.06 in GD vs. 3.00 ± 0.87 in HC, *P* = 0.0015, Fig. 2 a). However, expression of TACI was decreased in GD patients without statistical significance (mean MFI: 7.96 ± 4.06 in GD vs. 9.10 ± 3.37 in HC, *P* = 0.1285, Fig. 2b). No difference was observed in BR3 and TACI expression between HT patients and controls (Fig. 2b). These data suggested an altered expression of BAFF receptors on peripheral blood B lymphocytes in GD patients.

### 4. Altered expression of BAFF receptors was corrected by steroids in GO

Steroids have been shown to suppress serum BAFF concentrations in GO patients, however, its effects on the expression of BAFF receptors have not been investigated. We therefore analyzed serum BAFF concentrations, expression levels of BR3 and TACI on peripheral blood B lymphocytes before and at the end of steroids therapy in 10 GO patients. At baseline, there was no difference in serum BAFF levels and BAFF receptors expression between GO group and non-GO group (data were not shown). At the end of methylprednisolone treatment, the average serum BAFF concentration were decreased from 1.18 ± 0.27 ng/ml at baseline to 0.97 ± 0.10 ng/ml (*P* = 0.0364, Table 3). BR3 expression on peripheral blood B lymphocytes in GO patients was reduced (MFI was decreased from 6.26 ± 4.91 at baseline to 4.05 ± 1.58 at the end of therapy, *P* = 0.0083, Table 3). TACI expression in B lymphocytes was increased slightly after steroids therapy, but it was not statistically significant (MFI6.67 ± 1.96 at baseline vs. 7.05 ± 2.32 after steroids therapy, *P* = 0.1974, Table 3). These results indicated immunosuppressive effect of steroids on B lymphocytes was not only on the B cell surviving factor BAFF, but also on its receptors, mainly BR3.

**Table 3:** Effect of steroids treatment on BAFF and its receptors expression in GO patients

	GO baseline	Steroids therapy	*P*
BAFF (ng/ml)	1.18 ± 0.27	0.97 ± 0.10^*^	0.0364
BR3 MFI	6.26 ± 4.91	4.05 ± 1.58^**^	0.0083
TACI MFI	6.67 ± 1.96	7.05 ± 2.32	0.1974

*: vs. baseline, *P* < 0.05; **: vs. baseline, *P* < 0.01

## Discussions

In the present study, we observed elevated serum BAFF concentrations in patients affected with AITD. In addition, expression of BAFF receptors BR3 was increased on the peripheral B lymphocytes in GD patients. Steroids suppressed both BAFF expression and BR3 expression in GO patients.

Our data showed elevated serum BAFF levels in GD patients, in line with other studies describing increased serum BAFF levels in AITD [10, 12]. A potential role of BAFF in the occurrence of GD was supported by the investigation that blockade of BAFF through a BAFF specific receptor-Fc fusion protein improved hyperthyroidism in a murine model. There was no association between serum BAFF levels and TRAb titers in this study. A possible explanation is TRAbs measured in this study were antibodies with specific epitope as monoclonal antibody M22, but not the whole TRAb repertoire in the sera. Indeed, we found serum BAFF concentrations correlated positively with TPOAb titers in both GD and HT, an evidence supporting the relationship between BAFF and autoimmunity in AITD. Serum BAFF levels also correlated with free thyroxine concentrations in GD. Because over secretion of thyroxine is driven by TRAb in GD, our results indicate BAFF is important in TRAb production in GD.

There was an altered expression profile of BAFF receptors on peripheral blood B lymphocytes in GD patients in this study: BR3 expression was increased significantly while TACI expression was reduced without significance. BR3 expression on B lymphocytes was reduced in SLE patients but was increased in thyroid infiltrating lymphocytes in patients suffering from AITD [19]. Different results of TACI expression on peripheral B lymphocytes in SLE patients have been reported [5, 15]. The inconsistency indicates expression profiles of BAFF receptors may vary in different autoimmune diseases or disease status.[15]. BR3 and TACI oppositely regulate B cell homeostasis [2, 16, 23]: BR3 promotes B cells survival while TACI sensitizes B cells to apoptosis [23]. TACI also controls activity of B regulatory lymphocytes (Bregs) [16, 24], a subtype of B lymphocytes with immunosuppressive function. Loss of Bregs has been described in GD patients [25]. Therefore, the altered expression of BAFF and its receptors may mediate autoimmunity by enhancing BR3 signal pathway activity and inhibiting TACI signal pathway activity [2, 26, 27]. Unbiased blockade of both BR3 and TACI signal pathways will only augment inflammation due to decreased IL-10 secretion, as observed in multiple sclerosis (MS) patients [24]. To selectively inhibit the BR3 signal pathway and to restore the TACI signal pathway could be a better strategy to cure GD. Our data showed that there was no significant difference in the expression levels of BR3 and TACI in peripheral blood B lymphocytes between HT patients and healthy controls. The reasonable explanation is that HT is a T lymphocyte mediated autoimmune disease, the autoreactive B lymphocytes might impact the occurrence of the disease through an indirect effect.

Here we showed that the expression of BAFF was suppressed by steroids, which is consistent with previous studies. BR3 expression levels were decreased after steroids therapy. In large B cell lymphoma, BR3 rather than BAFF was an independent prognostic factor for steroids treatment [28]. Therefore, BR3 might be an important target of steroids in B lymphocytes. Due to the limited samples of patients receiving steroid treatment, it is necessary to explore the effect of steroids on TACI expression in a large patient sample in the future.

## Conclusions

In conclusions, altered expression of BAFF receptors was associated with autoimmunity of GD. Restoring the expression profile of BAFF receptors could be a new strategy to treat and cure GD.

## Data Availability

The datasets generated during and/or analyzed during the current study are available from the corresponding authors on reasonable request.

## Abbreviations

APRIL: A proliferation-inducing ligand
AITD: Autoimmune thyroid diseases
ATA: American Thyroid Association
BAFF: B lymphocyte activating factor
BR3: BAFF receptor 3
BCMA: B cell maturation antigen
BCR: B cell receptor
Bregs: Regulatory B cells
CAS: Clinical activity score
ECL: Electrochemiluminescence
ELISA: Enzyme linked immunosorbent assay
EUGOGO: European Group of Graves’ Orbitopathy
FITC: Fluorescein isothiocyanate
GD: Graves’ disease
GO: Graves’ orbitopathy
HC: Healthy controls
HT: Hashimoto’s thyroiditis
MFI: Mean fluorescent intensity
PBMC: Peripheral blood mononuclear cells
PE: Phycoerythrin
RA: Rheumatoid arthritis
SLE: Systemic lupus erythematosus
TACI: Transmembrane activator and calcium-modulating and cyclophilin ligand interactor
TIL: Thyroid infiltrating lymphocytes
TNF: Tumor necrosis factor
TPOAb: Thyroid peroxidase autoantibody
TRAb: Thyrotropin receptor autoantibody
